# Supplementary material for: From sequence to enzyme mechanism using multi-label machine learning
Source: BMC Bioinformatics. 2014 May 19;15:150. doi: 10.1186/1471-2105-15-150 (PMC4229970; doi:10.1186/1471-2105-15-150)
Supplement: Additional file 2 — Java code of ml2db. Additional file ml2db_code.tar.gz contains the Java source code to run the multi-label machine learning experiments and save the results to database. The code’s Javadoc is included. [file 1471-2105-15-150-S2.zip › additional file 2/ml2db/ml2db/doc/test/ml2db/mulan/learn/traintest/TrainTestEvaluatorTest.html]

TrainTestEvaluatorTest


JavaScript is disabled on your browser.


- Overview
- Package
- Class
- Use
- Tree
- Deprecated
- Index
- Help

- Prev Class
- Next Class

- Frames
- No Frames

- All Classes

- Summary:
- Nested |
- Field |
- Constr |
- Method

- Detail:
- Field |
- Constr |
- Method


test.ml2db.mulan.learn.traintest

## Class TrainTestEvaluatorTest

- java.lang.Object
- - junit.framework.Assert
  - - junit.framework.TestCase
    - - test.ml2db.mulan.learn.traintest.TrainTestEvaluatorTest

- All Implemented Interfaces:
  :   junit.framework.Test

  ---

    

  ```
  public class TrainTestEvaluatorTest
  extends junit.framework.TestCase
  ```

- - ### Constructor Summary

    Constructors

    Constructor and Description

    `TrainTestEvaluatorTest()`
  - ### Method Summary

    Methods

    | Modifier and Type | Method and Description |
    | --- | --- |
    | `static uk.ac.standrews.utils.main.database.TableRow` | `getEvaluationRow(TrainTestEvaluator evaluator, boolean predict, boolean saveTrueNegatives)` |
    | `static TrainTestEvaluator` | `getMulanTrainTestEvaluatorBRk1NN()` |
    | `static uk.ac.standrews.utils.main.database.TableRow` | `testCheckTrainTestEvalRow(uk.ac.standrews.utils.main.database.TableRow row)` Executes generic tests (number of metrics etc.) on a table row containing evaluation results. |
    | `void` | `testEvaluation1()` |
    | `static void` | `testEvaluationResults1(uk.ac.standrews.utils.main.database.TableRow row)` |

    - ### Methods inherited from class junit.framework.TestCase

      `countTestCases, getName, run, run, runBare, setName, toString`
    - ### Methods inherited from class junit.framework.Assert

      `assertEquals, assertEquals, assertEquals, assertEquals, assertEquals, assertEquals, assertEquals, assertEquals, assertEquals, assertEquals, assertEquals, assertEquals, assertEquals, assertEquals, assertEquals, assertEquals, assertEquals, assertEquals, assertEquals, assertEquals, assertFalse, assertFalse, assertNotNull, assertNotNull, assertNotSame, assertNotSame, assertNull, assertNull, assertSame, assertSame, assertTrue, assertTrue, fail, fail, failNotEquals, failNotSame, failSame, format`
    - ### Methods inherited from class java.lang.Object

      `equals, getClass, hashCode, notify, notifyAll, wait, wait, wait`

- - ### Constructor Detail


    - #### TrainTestEvaluatorTest

      ```
      public TrainTestEvaluatorTest()
      ```
  - ### Method Detail


    - #### testEvaluation1

      ```
      public void testEvaluation1()
      ```


    - #### getEvaluationRow

      ```
      public static uk.ac.standrews.utils.main.database.TableRow getEvaluationRow(TrainTestEvaluator evaluator,
                                                                  boolean predict,
                                                                  boolean saveTrueNegatives)
      ```


    - #### getMulanTrainTestEvaluatorBRk1NN

      ```
      public static TrainTestEvaluator getMulanTrainTestEvaluatorBRk1NN()
      ```


    - #### testCheckTrainTestEvalRow

      ```
      public static uk.ac.standrews.utils.main.database.TableRow testCheckTrainTestEvalRow(uk.ac.standrews.utils.main.database.TableRow row)
      ```

      Executes generic tests (number of metrics etc.) on a table row containing
      evaluation results.

      Parameters:
      :   `results` - a vector of table rows

      Returns:
      :   the first table row (for a train-test evaluation, only one row
          should be present)


    - #### testEvaluationResults1

      ```
      public static void testEvaluationResults1(uk.ac.standrews.utils.main.database.TableRow row)
      ```


- Overview
- Package
- Class
- Use
- Tree
- Deprecated
- Index
- Help

- Prev Class
- Next Class

- Frames
- No Frames

- All Classes

- Summary:
- Nested |
- Field |
- Constr |
- Method

- Detail:
- Field |
- Constr |
- Method
